# Loss of STAT1 in Bone Marrow-Derived Cells Accelerates Skeletal Muscle Regeneration

**DOI:** 10.1371/journal.pone.0037656

**Published:** 2012-05-23

**Authors:** Yan Gao, Yanfeng Li, Xing Guo, Zhenguo Wu, Wei Zhang

**Affiliations:** 1 Division of Life Science, The Hong Kong University of Science and Technology, Hong Kong, China; 2 Department of Anatomy, Capital Medical University, Peking, China; 3 Biomedical Research Institute, Shenzhen-PKU-HKUST Medical Center, Shenzhen, Guangdong, China; 4 Ji-Nan University–Hong Kong University of Science and Technology Joint Lab, College of Pharmacy, Ji-Nan University, Guangdong, China; Laboratory of Neuroendocrine-Immunology, Pennington Biomedical Research Center, United States of America

## Abstract

**Background:**

Skeletal muscle regeneration is a complex process which is not yet completely understood. Evidence suggested that the Janus kinase (JAK)–signal transducer and activator of transcription (STAT) pathway may have a role in myogenesis. In this study, we aim to explore the possible role of STAT1 in muscle regeneration.

**Methods:**

Wild-type and STAT1 knockout mice were used in this study. Tibialis anterior muscle injury was conducted by cardiotoxin (CTX) injection. Bone marrow transplantation and glucocorticoid treatment were performed to manipulate the immune system of the mice.

**Results:**

Muscle regeneration was accelerated in STAT1−/− mice after CTX injury. Bone marrow transplantation experiments showed that the regeneration process relied on the type of donor mice rather than on recipient mice. Levels of pro-inflammatory cytokines, TNFα and IL-1β, were significantly higher in STAT1−/− mice at 1 day and/or 2 days post-injury, while levels of anti-inflammatory cytokine, IL-10, were lower in STAT1−/− mice at 2 days and 3 days post-injury. Levels of IGF-1 were significantly higher in the STAT1−/− mice at 1 day and 2 days post-injury. Furthermore, the muscle regeneration process was inhibited in glucocorticoid-treated mice.

**Conclusions:**

Loss of STAT1 in bone marrow–derived cells accelerates skeletal muscle regeneration.

## Introduction

Skeletal muscle regeneration in response to trauma consists of three phases: the destruction phase, the repair phase and the remodeling phase [1], [2], [3]. The destruction phase is characterized by necrosis of myofibers, hematoma formation and the infiltration of inflammatory cells. Then, in the repair phase, the necrotic debris is phagocytosed, and satellite cells are activated to regenerate myofibers [4], [5]. For example, cardiotoxin (CTX) –induced injury caused an increase in MyoD expression in satellite cells at 2 days post-injury followed by an elevation of myogenin expression at 3 days post-injury [6], [7]. At last, in the remodeling phase, the regenerated myofibers mature and contract. The immune system plays a crucial role in muscle regeneration. Muscle injuries initiate a predictable series of responses by immune cells which are primarily myeloid cells such as neutrophils and macrophages [8], [9], [10]. These immune cells can be present in regenerative muscle at rather high concentrations [11], and are capable of releasing numerous soluble molecules, especially cytokines [12], [13], [14], that can affect the viability and transcriptional activities of regenerative muscle cells [15], [16].

The Janus kinase (JAK)–signal transducer and activator of transcription (STAT) pathway represents one of the best-characterized cellular signaling pathways in the immune system [17], [18]. Four JAKs (JAK1, 2, 3, and Tyk2) and seven STATs (STAT1, 2, 3, 4, 5a, 5b and 6) have been identified in the mouse and human genomes. The JAK–STAT pathway plays important roles in regulating cytokine signaling, which has been well established by the targeted disruption of genes encoding STATs [19]. Specifically, STAT1 is required for the expression of Interferon-regulated genes that are involved in innate immunity [20], [21], [22]. It remains unclear whether the JAK–STAT pathway plays an essential role in myogenesis. Several lines of evidence suggested that the JAK–STAT pathway may have a role in myogenic differentiation. STAT3 was found to be present in activated muscle satellite cells and proliferating myoblasts in regenerating rat muscles [23]. In response to leukemia inhibitory factor (LIF), proliferating primary myoblasts grown in culture were also found to contain higher levels of phosphorylated STAT3 [24], [25]. In addition, STAT3 was also shown to physically interact with MyoD [26]. In our previous study, we reported that the JAK1–STAT1–STAT3 pathway plays important roles in both proliferation and differentiation of myoblasts [27], and JAK2-STAT2 plays an opposite role during myogenic differentiation in comparison with JAK1 [28]. Down-regulation of either JAK1 or STAT1 by siRNA accelerates myogenic differentiation in both C2C12 cells and primary myoblasts [27].

Nevertheless, the aforementioned findings were mainly obtained by in vitro experiments, and none of these studies addressed the question of whether and how the JAK–STAT pathway is involved in muscle regeneration. In vivo, the direct microenvironments of the satellite cells and many regulatory factors play a major role in muscle regeneration [29], [30]. More importantly, it remains unclear whether the regulatory interactions between muscle and immune cells could be affected by JAK–STAT pathway during muscle regeneration. Therefore, in the present study, we took advantage of STAT1 knock-out mice to examine the role of JAK–STAT pathway in muscle regeneration. We found that muscle regeneration process was accelerated in the STAT1 knock-out mice after CTX injury. Through bone-marrow transplantation experiments, we also proved that loss of STAT1 in the myeloid cells contributes to the accelerated regeneration in STAT1 knock-out mice.

## Results

### Muscle regeneration was accelerated in STAT1−/− mice after CTX injury

**Figure 1:**
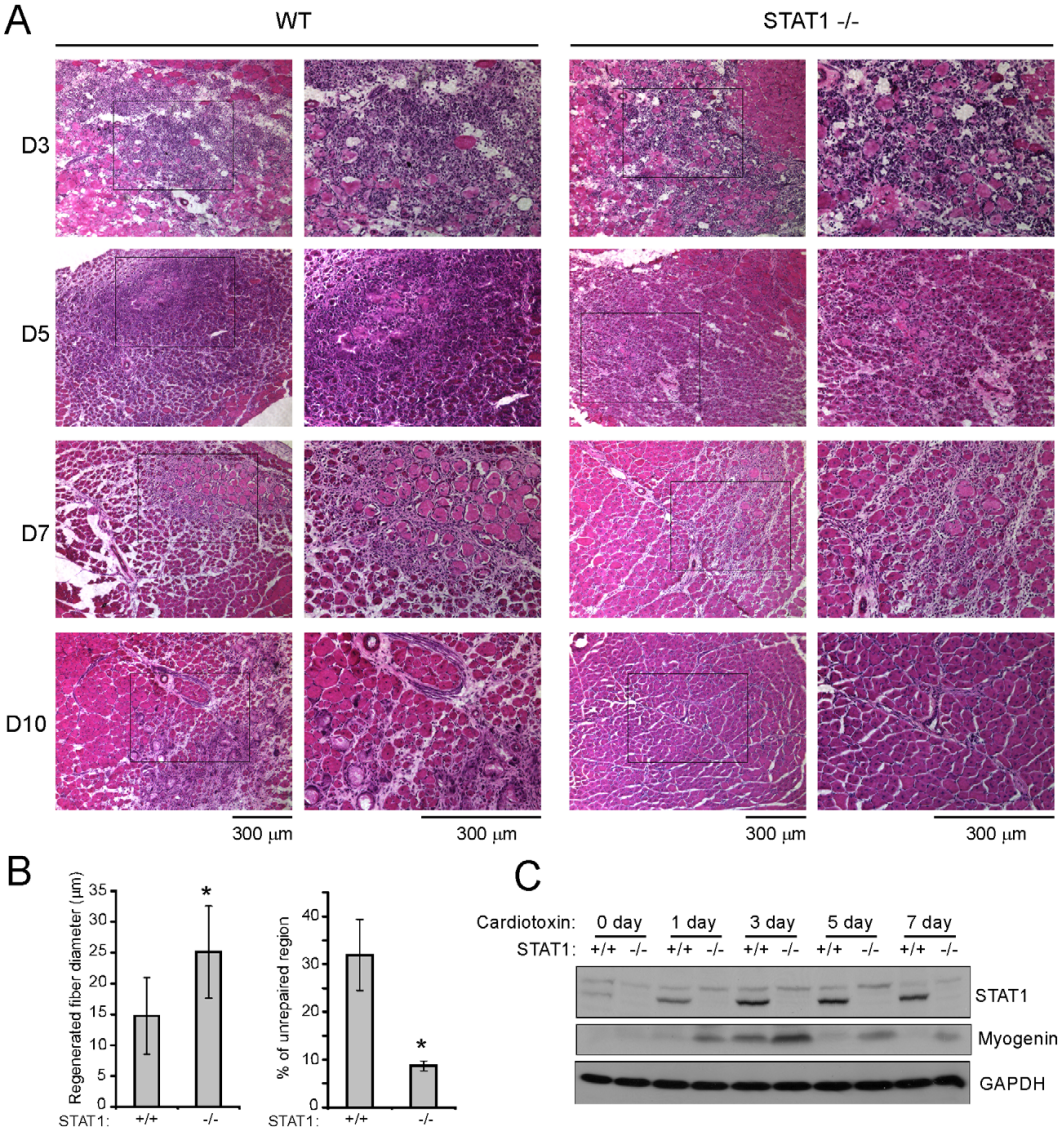
Muscle regeneration was accelerated in STAT1−/− mice after cardiotoxin injection. Tibialis anterior muscles from 2-month-old wild-type and STAT1−/− mice were injected with 30 ul of 10 nM cardiotoxin. The TA muscles were harvested at different time points as indicated after injury. (A) The muscles were fixed in 4% paraformaldehyde/PBS (pH 7.4) for 1 hour at 4°C, infiltrated sequentially with 10%, 20% and 30% sucrose, and embedded in O. C. T. solution. Six micrometers of cryosections were stained with hematoxylin and eosin. Bars: 300 um. (B) The diameter of the regenerated fibers and the percentage of un-repaired region from three independent experiments were measured 10 days post-injury. Data were presented as mean ± SD. Asterisk: p<0.05. (C) Tibialis anterior muscles were homogenized in protein lysis buffer. Equal amounts of protein were separated by SDS-PAGE followed by western blotting with different antibodies as indicated.

Previously, using muscle satellite cells as a model, we found that STAT1 was involved in the muscle differentiation process [27]. To further elucidate the function of STAT1 in vivo, we take advantage of the STAT1 knockout mice and use CTX injury model in our study. After CTX injection, muscle fibers will degenerate followed by activation, proliferation and differentiation of muscle satellite cells, resulting in the regeneration of muscle [6], [7]. We compared the regeneration process between the wild-type (WT) and STAT1 knockout (STAT−/−) mice. There was no obvious difference between WT and STAT1−/− mice before injury (Figure S1). At 3 days after CTX injection, massive infiltration of inflammatory cells was observed by H & E staining (Fig. 1A, upper panels), and there was no obvious difference between the two types of mice. At 5 days post-injury, more regenerating fibers can be found in the STAT1−/− mice than that in the WT mice, and the better regeneration state in STAT1−/− mice maintained through 7 days to 10 days post-injury (Fig. 1A, middle and lower panels). To obtain a quantitative comparison, we measured the diameter of the regenerated fiber and the percentage of un-repaired region between the two types of mice at 10 days post-injury. We found that the average diameter of the regenerated fibers was significantly larger, while the percentage of un-repaired region was significantly lower, in the STAT1−/− mice than that in the WT mice (Fig. 1B). H & E staining of the uninjected contralateral TA muscles was shown in Supplementary Figure 1. Myogenin is a muscle differentiation marker with the highest expression levels at 3 days after CTX injury [6], [7], and the expression levels of Myogenin were much higher in STAT1−/− mice than that in WT mice, suggesting that regeneration process was accelerated in the STAT1−/− mice (Fig. 1C). Interestingly, expression of STAT1 in WT mice was also upregulated upon injury with the peak at 3 days post-injury (Fig. 1C), which is corresponding to the number of infiltrated inflammatory cells.

### Muscle satellite cells from STAT1−/− and WT mice show similar differentiation ability in vitro

Our previous work showed that knockdown of STAT1 by siRNA in vitro promoted muscle satellite cell differentiation [27]. Thus, we hypothesized that the accelerated muscle regeneration in STAT1−/− mice in vivo may be due to the increased differentiation ability of STAT1−/− muscle progenitor cells. So we isolated the muscle satellite cells from WT and STAT1−/− mice, and compared their differentiation ability in vitro. To our surprise, neither the expression levels of Myosin heavy chain (MHC) and Myogenin nor the morphology of the satellite cells showed obvious difference between the two types of mice (Figure S2). These data suggest that, under in vitro culture condition, muscle satellite cells from STAT1−/− mice have no increased differentiation ability compared with that from WT mice.

### Bone marrow derived cells contribute to the accelerated muscle regeneration in STAT1−/− mice

Muscle regeneration process is mainly achieved through the activation, proliferation and differentiation of muscle progenitor cells. Our above data showed that, under the same in vitro culture environment, muscle satellite cells from STAT1−/− mice don't exhibit stronger differentiation potential over WT mice. So we deduced that the in vivo environment after injury might not be the same between the two types of mice. Since inflammatory cells are the main factors affecting the local environment at the injury sites and STAT1 is known to be an important factor in the signaling of immune response, we wonder if the different properties of the inflammatory cells from the two types of mice may contribute to the accelerated regeneration in STAT1−/− mice.

**Figure 2:**
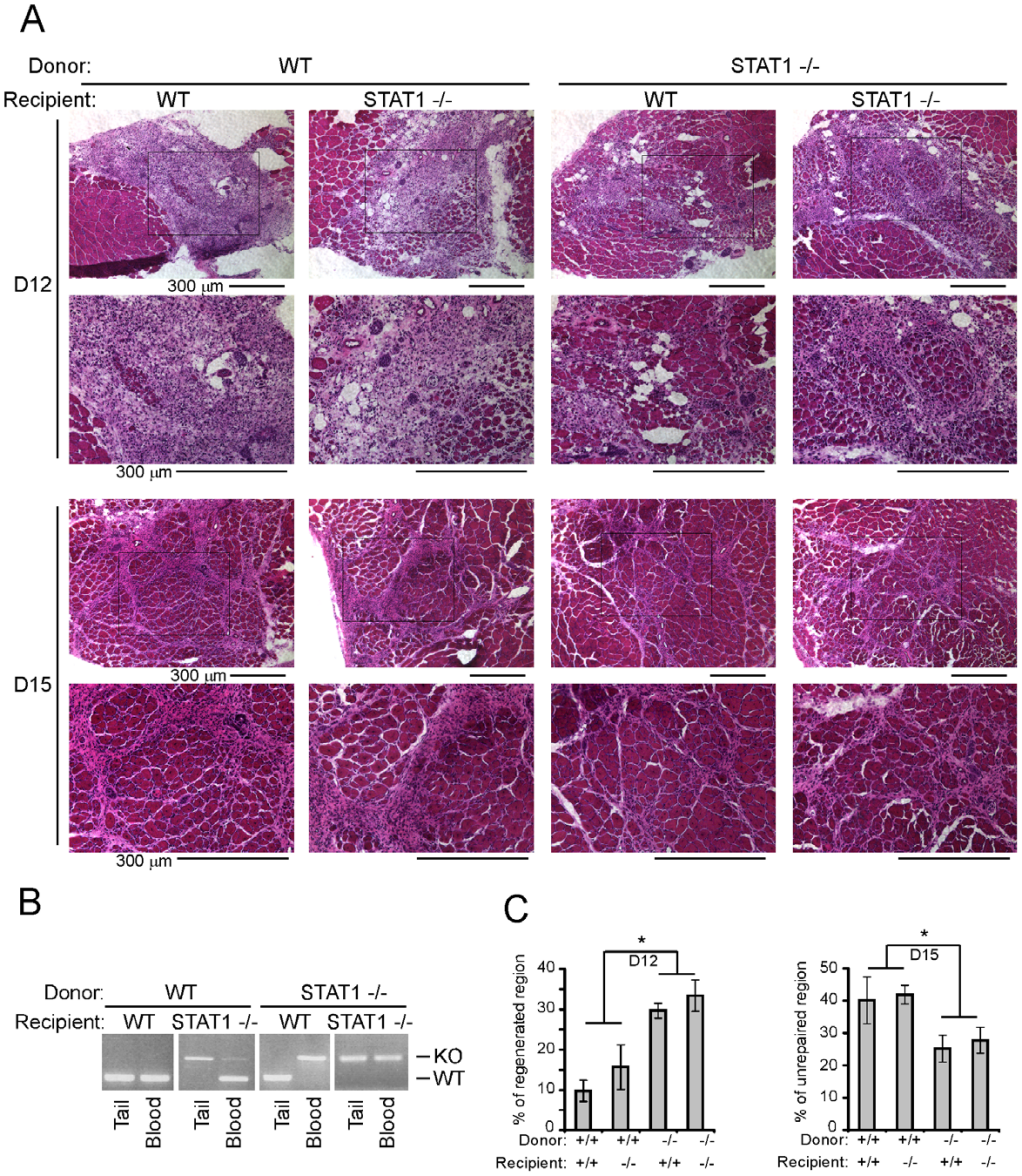
Loss of STAT1 in the bone marrow-derived cells accelerated muscle regeneration. Donor bone marrow cells were freshly prepared from the femurs of donor mice and filtered through a sterile 40 µm nylon Cell Strainer to remove debris. The cells were suspended in DMEM without serum prior transplantation. The recipient mice were given 8 Gy Gamma-irradiation and each injected with 2×10^6^ donor bone marrow cells through retro-orbital vein. Six to eight weeks after bone marrow transplantation, tibialis anterior muscles of the recipient mice were injected with 30 ul of 10 nM cardiotoxin. (A) The muscles were fixed in 4% paraformaldehyde/PBS (pH 7.4) for 1 hour at 4°C, infiltrated sequentially with 10%, 20% and 30% sucrose, and embedded in O. C. T. solution. Six micrometers of cryosections were stained with hematoxylin and eosin. Bars: 300 um. (B) The tails and peripheral blood cells from the recipient mice were harvested, and genomic DNA was extracted. PCR was used for genotyping. The upper and lower bands indicate genotypes of STAT1−/− and wild-type respectively. (C) The percentage of regenerated region 12 days post-injury and unrepaired region 15 days post-injury from three independent experiments were measured. Data were presented as mean ± SD. Asterisk: p<0.05.

To test this, we performed the bone marrow transplantation experiment. First, the recipient mice received gamma-irradiation to eliminate their own bone marrow cells. Then, fresh bone marrow cells from either STAT1−/− or WT donor mice were transplanted to the recipient mice. Six weeks after bone marrow transplantation, the bone marrow cells from the recipient mice will be mainly originated from the donor mice (Fig. 2B). The recipient mice were then subjected to CTX injury. Since a certain part of the muscle stem cells were also killed by gamma-irradiation, the regeneration process will be generally slow in these recipient mice, and observable regenerated fibers can be seen around 12 days post-injury. When STAT1−/− mice serve as donor mice, regardless of the type of recipient mice, muscle regeneration was better than when WT mice serve as donor (Fig. 2A, compare the left two columns with the right two columns). Two types of recipient mice with the same donor mice exhibit similar regeneration speed (Fig. 2A, compare the first with the second column, or the third with the forth column). The difference among the four groups was further quantified by measuring the percentage of regenerated region at 12 days post-injury and the percentage of un-repaired region at 15 days post-injury (Fig. 2C). The above data suggest that the origin of the bone marrow cells might be the key factor determining the speed of regeneration, and muscle progenitor cells from STAT1−/− and WT mice behave similarly under the same in vivo environment.

### STAT1−/− and WT mice have similar extent of macrophage infiltration, but distinct profile of cytokines, at the injury site after CTX injection

**Figure 3:**
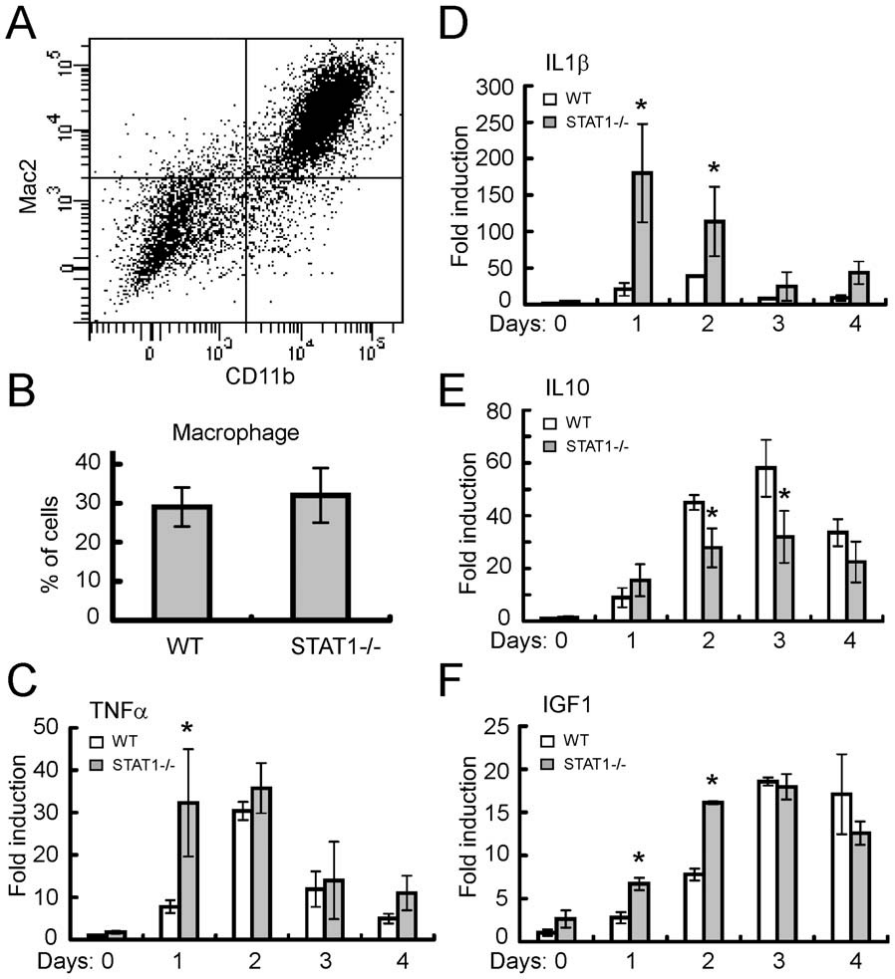
STAT1−/− and WT mice have similar extent of macrophage infiltration, but distinct profile of cytokines, after cardiotoxin injection. (A) Tissues from injured tibialis anterior, Gastrocnemius and Soleus muscles were digested twice in DMEM containing 0.2% Collagenase B and 0.2% Trypsin-EDTA at 37°C for 45 min. The digested tissues were applied to Ficoll-Paque centrifugation and the interface band was collected. For each staining reaction, 10^6^ cells were stained with PE-Cy5-anti-CD11b and PE-anti-Mac2 in 100 ul system and subjected to flow cytometry analysis. (B) The percentage of macrophages from three independent experiments was analyzed. Data were presented as mean ± SD. (C–F) Total RNA was isolated from tibialis anterior muscles and reverse transcribed. Triplicate samples were subjected to quantitative PCR. GAPDH was used as an internal control. The relative abundance of genes of interest was calculated after normalization to GAPDH. Data from three independent experiments were presented as mean ± SD. Asterisk: p<0.05.

The above evidence leads us to think that the accelerated regeneration in the recipient mice transplanted with STAT1−/− bone marrow should be due to the distinct local environment at the injury site. Massive infiltration of macrophages occurs after CTX-induced injury, which are indispensable for the degeneration and regeneration of muscle [31]. We therefore examined macrophages by FACS analysis in our study. Two macrophage markers, Mac1 (CD11b) and Mac2 were used to define macrophage population (Fig. 3A). In this experiment, the single nuclear cells from the injured muscle tissue at 2 days post-injury were analyzed by FACS to measure the percentage of macrophages. In terms of the percentage of macrophages over total single nuclear cells, we didn't find obvious difference between STAT1−/− and WT mice (Fig. 3B).

We extracted the RNA from the injured muscle and measured the cytokine profile using real-time PCR. Levels of pro-inflammatory cytokines such as TNFα and IL-1β were significantly higher in STAT1−/− mice at 1 day and/or 2 days post-injury, while anti-inflammatory cytokines such as IL-10 were lower in STAT1−/− mice at 2 days and day 3 post-injury (Fig. 3C, 3D and 3E). More importantly, a well-known growth factor which can promote the proliferation and differentiation of muscle satellite cells, IGF-1 [32], [33], has much higher expression levels in STAT−/− mice at 1 day and 2 days post-injury (Fig. 3F). This is consistent with our finding that regeneration process was accelerated in STAT1−/− mice.

### Muscle regeneration was inhibited in glucocorticoid treated mice

**Figure 4:**
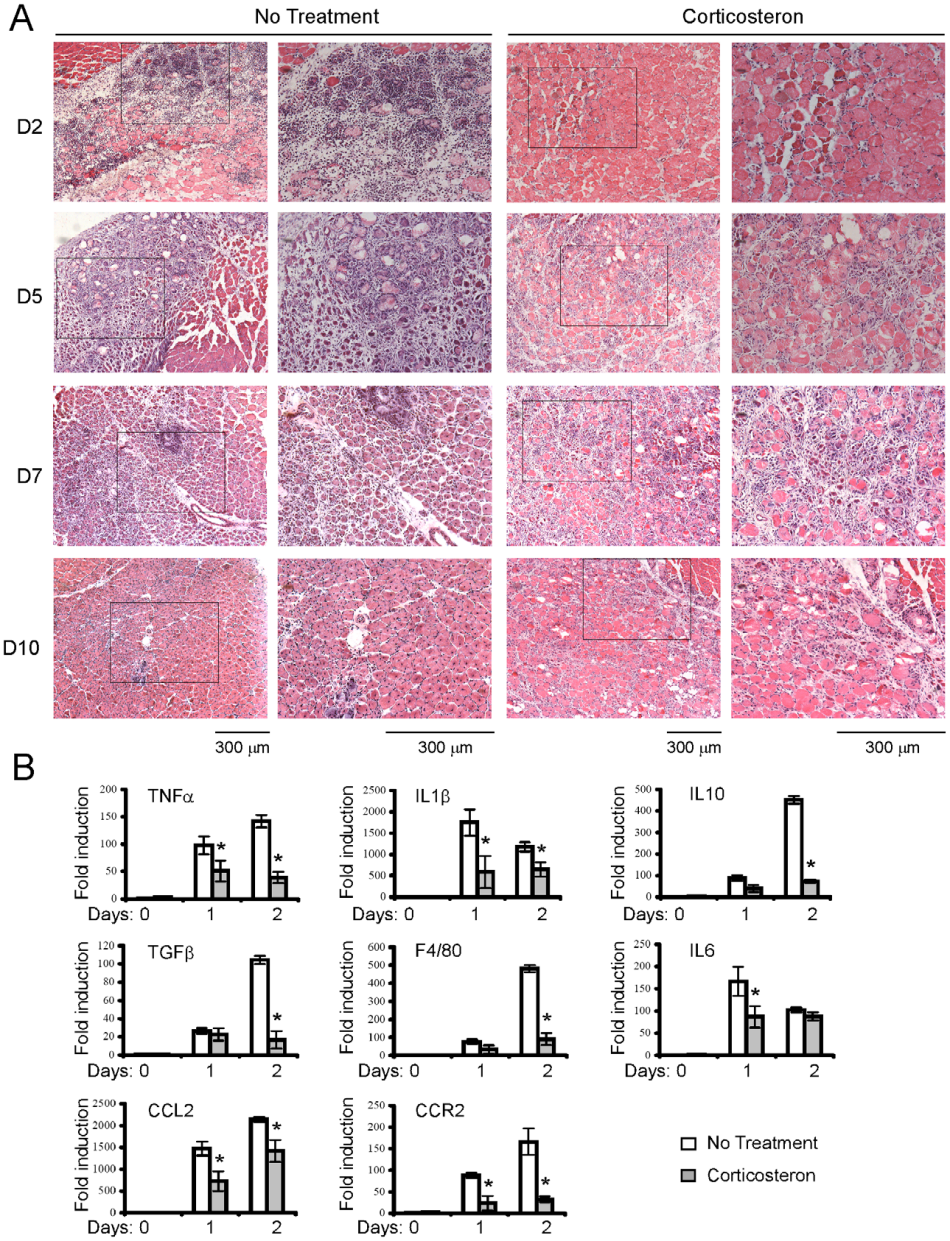
Muscle regeneration was inhibited in glucocorticoid treated mice. Mice were treated with 0.9% saline drinking water supplemented with 100 ug/ml corticosterone starting from 3 days prior cardiotoxin injury until the mice were sacrificed. Tibialis anterior muscles from corticosterone treated and untreated wild-type mice were injected with 30 ul of 10 nM cardiotoxin. The tibialis anterior muscles were harvested at different time points as indicated after injury. (A) The muscles were fixed in 4% paraformaldehyde/PBS (pH 7.4) for 1 hour at 4°C, infiltrated sequentially with 10%, 20% and 30% sucrose, and embedded in O. C. T. solution. Six micrometers of cryosections were stained with hematoxylin and eosin. Bars: 300 um. (B) Total RNA was isolated from tibialis anterior muscles and reverse transcribed. Triplicate samples were subjected to quantitative PCR. GAPDH was used as an internal control. The relative abundance of genes of interest was calculated after normalization to GAPDH. Data from three independent experiments were presented as mean ± SD. Asterisk: p<0.05.

The increased levels of the pro-inflammatory cytokines in STAT1−/− mice suggest that local inflammation is more severe in STAT1−/− mice at 1 to 2 days post-injury. This is consistent with previous report [31]. Then we hypothesized that alleviated inflammation may slow down the process of muscle regeneration. So we treated the mice with corticosterone, one type of glucocorticoid, to suppress immune response, and applied CTX-induced injury to these mice. The color of the newly dying fibers is lighter than uninjured or repaired muscle fibers. We can find that most of the fibers (2 days post-injury) in the corticosterone-treated mice are actually dying fibers, rather than normal fibers. As expected, infiltration of inflammatory cells was greatly reduced in these mice after injury. Correspondingly, most of the necrotic fibers haven't been removed by phagocytic cells until 10 days post-injury, and regeneration process was much delayed (Fig. 4A). The expression levels of both pro-inflammatory and anti-inflammatory cytokines were significantly lower in the mice treated with corticosterone (Fig. 4B). These data were consistent with previous findings that reduced numbers of leukocytes prior to muscle injury by toxin injection slowed both the removal of cellular debris and muscle regeneration [31], [34], and provided further evidence that infiltration of inflammatory cells and secretion of inflammatory cytokines are necessary for the proper regeneration process.

## Discussion

We have previously explored the role of JAK-STAT pathway in the muscle differentiation process, and showed that primary myoblasts transfected with STAT1-siRNA differentiated faster [27]. In the present study, we found that muscle regeneration process was accelerated in the STAT1 knock-out mice after CTX injury. However, the underlying mechanisms for the in vitro and in vivo effects of STAT1 are different. Although transient transfection of muscle satellite cells with STAT1 siRNA obviously promoted muscle differentiation, isolated myoblasts from STAT1−/− and WT mice exhibited no difference on the differentiation process. Thus, the accelerated muscle regeneration in STAT1−/− mice may be caused by the STAT1-deficient inflammatory cells. This hypothesis was proved by our bone transplantation experiment. Since inflammatory cells are mainly derived from bone marrow, depletion of bone marrow cells from the recipient mice by irradiation followed by transplantation of bone marrow from donor mice proved to be an effective strategy to investigate the potential role of inflammatory cells. When we applied bone marrow transplantation to the mice, the regeneration process was found to rely on the type of donor mice rather than on recipient mice, suggesting that, under similar inflammation state, STAT1−/− and WT muscle satellite cells behave similarly. This is consistent with our aforementioned data which showed that isolated myoblasts from STAT1−/− and WT mice differentiated at similar levels.

Inflammatory cells are capable of releasing numerous soluble molecules, especially cytokines [12], [13], [14], that can affect the viability and transcriptional activities of regenerative muscle cells [15], [16]. In our case, although the number of infiltrated inflammatory cells didn't obviously increase in STAT1−/− mice, the cytokines secreted by these inflammatory cells (e.g., TNFα and IL-1β) were significantly higher in STAT1−/− mice at early time points (1 day or 2 days after CTX injury), suggesting that the expression of these inflammatory factors may be regulated directly or indirectly by STAT1. The expression of STAT1 in WT mice was upregulated upon-injury, which was thought to reflect the number of infiltrated inflammatory cells. Upon injury, muscle fibers undergo degeneration and regeneration. Damaged muscle debris need to be removed by inflammatory cells (e.g., neutrophils and macrophages). Mice with slower rates of phagocytic removal of muscle debris showed slower rates of muscle regeneration [31], [34], [35]. Consistent with previous findings, in our study, we found that accelerated muscle regeneration in STAT1−/− mice correlates to the increased inflammation. This should be the main reason why STAT1−/− mice has better regeneration after injury.

The contribution of inflammation to muscle regeneration was further examined by glucocorticoid treatment in our study. Glucocorticoid has been clinically used for many years to suppress immune response. The number of infiltrated inflammatory cells was greatly reduced after CTX injury in the glucocorticoid treated mice, followed by the failure of debris removal and muscle regeneration, suggesting that glucocorticoid suppressed both the infiltration and the function of inflammatory cells. Reducing the numbers of phagocytic leukocytes from mice prior to muscle injury by toxin injection slows both the removal of cellular debris and muscle regeneration [31], [34]. In vivo, macrophage suppression leads to incomplete skeletal muscle regeneration [36]. Prevention of monocyte recruitment to the site of injury completely inhibits skeletal muscle regeneration [31]. This could be phiological significant, implicating that immuno-supressed patients may suffer from slowed muscle regeneration.

Moreover, cytokines secreted by inflammatory cells (e.g., IGF-1) are also necessary for muscle satellite cells to be activated, proliferate and differentiate [1], [37], [38]. In particular, IGF-1 is critical for skeletal muscle growth [32], [33]. In vitro, IGF-1 is able to alter the expression of myogenic regulatory factors and promote the proliferation and the differentiation of myoblasts [1], [39]. In our study, levels of IGF-1 were significantly higher in the STAT1−/− mice after injury. This partially explained why muscle regeneration was accelerated in STAT1−/− mice. Overall, our findings provide a possible target for modulation of muscle regeneration. Down-regulation of STAT1, or increasing the reactivity of the immune system, may contribute to the accelerated muscle regeneration, which povide a practical application for our study.

On the other hand, there have been controversial reports regarding the function of STAT1 in immune-modulation. For example, patients with diabetes, which is a proinflammatory environment, suffered form impaired wound healing. In our study, proinflammatory cytokines are actually upregulated in the STAT1 −/− mouse after injury, which is not uncommon in the literature, yet somewhat still controversial. A possilbe explanation for such paradoxical findings could be that many other factors in different microenviroment control the function of STAT1. Moreover, although it is well known that STAT1 is an important transcriptional factor in the signaling of immune response, it is still not clear in our study how STAT1 regulates the expression of the inflammatory cytokines and chemokines. In the future, it would be conceivably beneficial to extensively elucidate the regulation of STAT1 on these factors.

## Materials and Methods

### Mice and animal care

STAT1 knockout mice (C57BL/6J strain) were purchased from The Jackson Laboratory. All mice were maintained in the animal center at the Hong Kong University of Science and Technology. The mice were kept at a constant temperature (20°C) with a light cycle of 12∶12h, and fed with normal chow. All animals were handled in accordance with the guidelines of the Administrative Panel on Laboratory Animal Care of the Hong Kong University of Science and Technology. The study was approved by the Administrative Panel on Laboratory Animal Care of the Hong Kong University of Science and Technology.

### Antibodies

Antibody against STAT1 was from Upstate Biotechnology. Anti-myogenin and anti-Myosin heavy chain (MHC) were purchased from Developmental Studies Hybridoma Bank. Anti-Actin and GAPDH were from Santa Cruz. PE-Cy5-anti-CD11b, PE-anti-Mac2 and FITC-anti-F4/80 were from eBioscience.

### Induction of muscle injury, histochemistry and immunohistochemistry

Muscle injury was conducted as previously described with slight modifications [7]. Tibialis anterior (TA) muscles from 2-month-old wild-type and STAT1 konckout mice were injected with 30 µl of 10 nM cardiotoxin (CTX, Cal-biochem). The injected TA muscles were harvested at different time points after injury, with uninjected contralateral TA muscles as control. For hematoxylin and eosin (H & E) staining, the muscles were fixed in 4% paraformaldehyde/PBS (pH 7.4) for 1 hour at 4°C, infiltrated sequentially with 10%, 20% and 30% sucrose, and embedded in O. C. T. solution (Sakura). For immunohistochemistry, the TA muscles were embedded without fixation. Six micrometers of cryosections were processed for histological and immunofluorescence analysis. The diameter of regenerated fiber and the area of regenerated and unrepaired region were measured by Image 2.0 software. Quantification from three seperated experiments was shown as mean ± SD.

### Myoblasts isolation

Myoblasts isolation was performed as described previously with minor modifications [27]. In brief, skeletal muscles of 2-week-old mice were isolated, minced, and digested in 1.25 mg/ml protease type XVII (Sigma-Aldrich) for 1 h at 37°C. Fibroblasts were removed by pre-sedimentation. Satellite cells were generated by culture in F10 medium (Invitrogen) supplemented with 20% FBS in culture dishes coated with 4 mg/ml Matrigel (BD Biosciences). To observe in vitro differentiation, myoblasts were induced in DM (DMEM with 5% horse serum) to differentiate.

### RNA and protein analysis

Total RNA was isolated from uninjured or injured TA muscles using TRIzol reagent (Invitrogen). The first strand cDNA was synthesized with the ImProm-II reverse transcription system (Promega). Quantitative PCR was performed with 2 µl of cDNA and 1 unit of Taq polymerase in 25-µl reactions. 2× SYBR Green Supermix from Bio-Rad was used to set up 25 µl real-time PCR reactions according to the manufacturer's instructions. Triplicate samples were subjected to quantitative PCR using a Stratagene Mx3000P real-time PCR system with the maximum cycle number of 40. GAPDH was used as an internal control. The relative abundance of genes of interest was calculated after normalization to GAPDH. The primers are: TNFα, forward: 5′ TTC CAG ATT CTT CCC TGA GGT and reverse: 5′ TAA GCA AAA GAG GAG GCA ACA; IL1β, forward: 5′ TGA CGT TCC CAT TAG ACA ACT G and reverse: 5′ CCG TCT TTC ATT ACA CAG GAC A; IL10, forward: 5′ ACC AGC TGG ACA ACA TAC TGC and reverse: 5′ TCA CTC TTC ACC TGC TCC ACT; IGF1, forward: 5′ ACA GCT GGA CCA GAG AC and reverse: 5′ ACA GTA CAT CTC CAG TC; GAPDH, forward: 5′ CCC ACT CTT CCA CCT TCG and reverse: 5′ TCC TTG GAG GCC ATG TAG GCC AT. Data from three independent experiments were analyzed by student t-test, and p<0.05 was considered statistically significant. For protein analysis, TA muscles were homogenized in protein lysis buffer (50 mM HEPES [pH 7.6], 10% glycerol, 1% Triton X-100, 150 mM NaCl, 1 mM EGTA, 1.5 mM MgCl2, 100 mM NaF, 20 mM ρ-nitrophenyl phosphate, 20 mM glycerolphosphate, 2 mM dithiothreitol, 50 µM sodium vanadate, 0.5 mM henylmethylsulfonyl fluoride, 2 µg/ml aprotinin, 0.5 µg/ml leupeptin, and 0.7 µg/ml pepstatin). Equal amounts of protein were separated by SDS-PAGE followed by western blotting with different antibodies.

### Isolation of inflammatory cells and FACS analysis

Total inflammatory cells were isolated as described [31]. Briefly, Tissues from injured TA, Gastrocnemius and Soleus muscles were digested twice in DMEM containing 0.2% Collagenase B (Roche) and 0.2% Trypsin-EDTA at 37°C for 45 min. The digested tissues were applied to cell strainer (40 µm Nylon) to remove muscle fibres. To get mononuclear cells, the cells were resuspended in 4 ml PBS and overlayed on top of 3 ml Ficoll-Paque Plus (GE, 17-1440-02/03) in a 14 ml round bottom tube, and then centrifuged at 450 g for 30 min. The interface band was transfered into a 15 ml centrifuge tube, washed with PBS and resuspended in PBS containing 1% FBS for the following experiments. For each staining reaction, 10^6^ cells were stained in 100 ul system with the optimized amount of antibodies. The cells were incubated at 4°C in the dark for 1 hour and washed twice with PBS. For live cell sorting, the stained cells were directly subjected to flow cytometer. For FACS analysis, Mac1 (CD11b) and Mac2 were used as monocyte/macrophage markers.

### Bone marrow transplantation

Bone marrow transplantation was performed as described [40]. Donor bone marrow cells were freshly prepared from the femurs of donor mice and filtered through a sterile 40 µm nylon Cell Strainer (Falcon) to remove debris. The cells were suspended in DMEM (Gibco) without serum prior transplantation. The recipient mice were given acidified water one week before irradiation and were kept feeding with acidic water until sacrificed. The day before transplantation, the recipient mice were given 8 Gy Gamma-irradiation (^137^Cesium Gammacell source). On the day of transplatation, the recipient mice were anaesthetized and each injected with 2×10^6^ donor bone marrow cells through retro-orbital vein. Six to eight weeks after bone marrow transplantation, the recipient mice were subjected to CTX injury.

### Glucocorticoid treatment

Glucocorticoid treatment was conducted as previously described [41]. Briefly, WT mice were treated with 0.9% saline drinking water supplemented with 100 ug/ml corticosterone (Sigma, St. Louis, MO) starting from 3 days prior CTX injury until the mice were sacrificed at certain days post injury. Water consumption was monitored and there is no difference between the treated mice and the control mice (around 5–7 ml/day) during the period of experiment.

### Statistical analysis

Statistical analysis was performed by SPSS 11.0 software. For H & E staining, data from three independent experiments were analyzed by One-way ANOVA, and multiple-comparison was performed to compare data from four groups. For cytokine production, data from three independent experiments were analyzed by student t-test. P values less than 0.05 was considered statistically significant.

## Supporting Information

Figure S1 **H & E staining of the uninjured TA muscles.** Uninjected contralateral TA muscles from WT and STAT1−/− mice were fixed and subjected to H & E staining.
(TIF)

Figure S2 **Muscle satellite cells from STAT1−/− and WT mice show similar differentiation ability in vitro.** Skeletal muscles of 2-week-old mice were isolated, minced, and digested in 1.25 mg/ml protease type XVII for 1 h at 37°C. Fibroblasts were removed by pre-sedimentation. Satellite cells were generated by culture in F10 medium supplemented with 20% FBS in culture dishes coated with 4 mg/ml Matrigel. To observe in vitro differentiation, myoblasts were induced in DM (DMEM with 5% horse serum) to differentiate. (A) Whole cell extracts were separated by SDS-PAGE followed by western blotting with different antibodies as indicated. (B) Cells were fixed at DM12h, and phase-contrast images were presented. DM: differentiation medium.
(TIF)
