# Supplementary figures and images for: Loss of STAT1 in Bone Marrow-Derived Cells Accelerates Skeletal Muscle Regeneration
Source: PLoS One. 2012 May 23;7(5):e37656. doi: 10.1371/journal.pone.0037656 (PMC3359303; doi:10.1371/journal.pone.0037656)

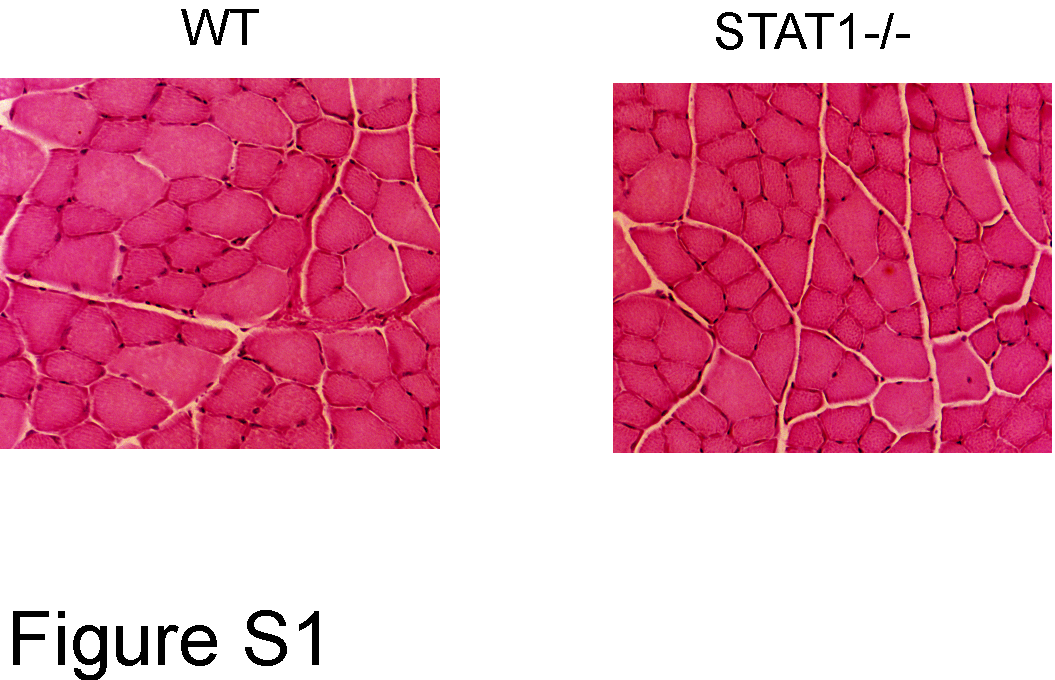

Supplement: Figure S1 — H & E staining of the uninjured TA muscles. Uninjected contralateral TA muscles from WT and STAT1−/− mice were fixed and subjected to H & E staining. (TIF) [file pone.0037656.s001.tif]

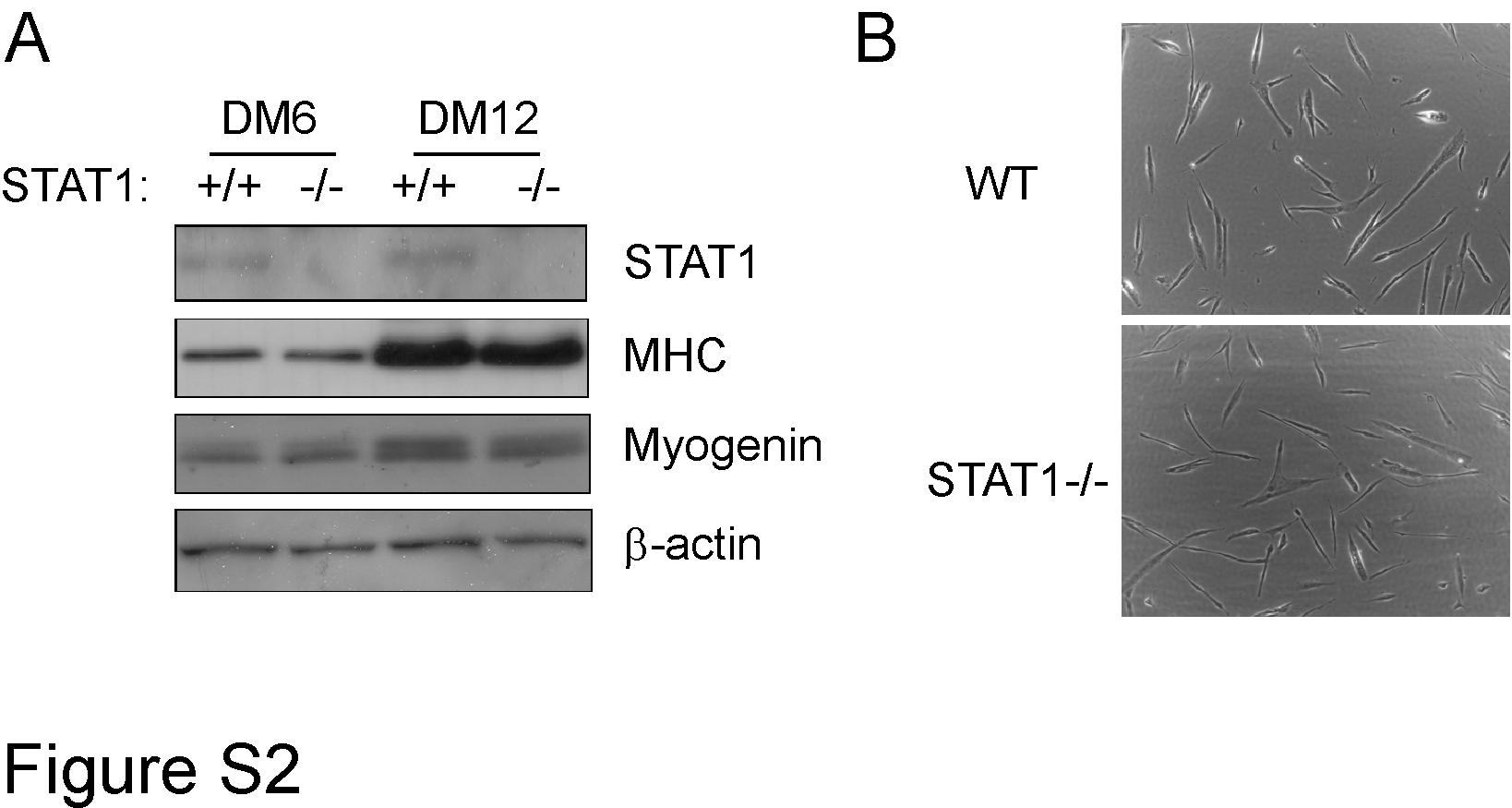

Supplement: Figure S2 — Muscle satellite cells from STAT1−/− and WT mice show similar differentiation ability in vitro. Skeletal muscles of 2-week-old mice were isolated, minced, and digested in 1.25 mg/ml protease type XVII for 1 h at 37°C. Fibroblasts were removed by pre-sedimentation. Satellite cells were generated by culture in F10 medium supplemented with 20% FBS in culture dishes coated with 4 mg/ml Matrigel. To observe in vitro differentiation, myoblasts were induced in DM (DMEM with 5% horse serum) to differentiate. (A) Whole cell extracts were separated by SDS-PAGE followed by western blotting with different antibodies as indicated. (B) Cells were fixed at DM12h, and phase-contrast images were presented. DM: differentiation medium. (TIF) [file pone.0037656.s002.tif]
